# Impaired interhemispheric synchrony in Parkinson’s disease with depression

**DOI:** 10.1038/srep27477

**Published:** 2016-06-06

**Authors:** Yajing Zhu, Xiaopeng Song, Mingze Xu, Xiao Hu, Erfeng Li, Jiajia Liu, Yonggui Yuan, Jia-Hong Gao, Weiguo Liu

**Affiliations:** 1 Department of Neurology, Affiliated Brain Hospital of Nanjing Medical University, Nanjing 210029, China; 2 Department of Biomedical Engineering, College of Engineering, Peking University, Beijing 100871, China; 3 Department of Psychiatry and Psychosomatics, Affiliated ZhongDa Hospital of Southeast University, The Institute of Neuropsychiatry of Southeast University, Nanjing 210009, China; 4 Center for MRI Research, Beijing City Key Lab for Medical Physics and Engineering, McGovern Institution for Brain Research, Peking University, Beijing 100871, China

## Abstract

The alterations of interhemispheric resting-state functional connectivity (FC) in Parkinson’s disease (PD) with depression remain unclear, so we aimed to explore the differences of interhemispheric FC between PD with and without depression. Twenty-one depressed PD (DPD) patients, 49 non-depressed PD (NDPD) patients and 50 matched healthy controls (HC) participated in this study. Resting-state functional magnetic resonance imaging (fMRI) data were analyzed with the voxel-mirrored homotopic connectivity (VMHC) approach. The DPD patients showed lower VMHC values in the bilateral dorsolateral prefrontal cortex (DLPFC) and calcarine cortex compared to both NDPD and HC groups, and further receiver operating characteristic curves (ROC) analyses revealed that the VMHC in these two brain areas could be used as biomarkers to distinguish DPD from NDPD and from HC. The pooled PD patients (both DPD and NDPD) exhibited decreased VMHC in the bilateral putamen, middle occipital gyrus (MOG), postcentral gyrus (PoCG), paracentral lobule (PCL) and cerebellum posterior lobe when compared with HC. Decreased VMHC values within the DLPFC and calcarine cortex appeared to be unique features for DPD and might be used as potential neuroimaging markers to distinguish DPD patients from NDPD and HC groups. These findings may underlie the neural mechanisms of depression in PD.

Parkinson’s disease (PD) is the second most common neurodegenerative disorder, affecting more than 1% of the elderly^1^, and is characterized by classic motor symptoms, including resting tremor, rigidity, bradykinesia and postural instability^2^. Recently, growing evidence has shown that numerous non-motor symptoms occur in PD patients and depression is one of them, affecting approximately 40–50% of the patients^3^,^4^,^5^. In addition to the unpleasant mood characteristics, depression may also be accompanied by severe disability, such as cognitive attenuation, impaired sensory perceptual functions^6^, and decreased quality of life^5^,^7^. However, the pathophysiology of depression in PD patients is not fully understood.

During the last decade, many structural and functional neuroimaging studies have attempted to explore the possible mechanisms of depression in PD^8^,^9^,^10^,^11^,^12^,^13^,^14^,^15^,^16^. Structural neuroimaging studies showed that PD patients with depression displayed grey matter loss and white matter reduction in the prefrontal, temporal and some limbic regions^8^,^9^,^10^. Functional neuroimaging studies also reported decreased glucose metabolism or regional cerebral blood flow (rCBF) in the dorsolateral prefrontal cortex (DLPFC), orbitofrontal cortex, inferior and medial frontal gyrus, anterior cingulate cortex, thalamus, and other limbic regions in depressed PD patients^11^,^12^,^13^,^14^. Previous functional MRI (fMRI) studies have demonstrated the association between depression in PD and abnormal activities in the subgenual cingulate, orbitofrontal and temporoparietal cortices^15^,^16^. These previous findings suggested that depression in PD might be associated with abnormal alterations in wide-spread subcortical-cortical areas, especially the impaired cognitive control of the frontal lobe over the subcortical limbic areas.

Apart from the above techniques, the interhemispheric resting-state functional connectivity (FC), however, is still an unexplored aspect of neuroimaging characteristics for depression in PD. Homotopic functional connectivity, which represents the functional architecture for the synchrony of spontaneous brain activities between geometrically corresponding regions in each hemisphere, is one of the most salient characteristics of the brain’s intrinsic functional architecture, and may reflect the importance of interhemispheric communication to integrated brain function underlying coherent cognition and behavior^17^. PD is usually of unilateral motor onset, and the asymmetry in clinical presentation on the two sides of body could reflect the imbalance in the pathological progression in the two hemispheres^18^,^19^. Considering that previous studies have shown decreased interhemispheric FC in major depressive disorder (MDD)^20^,^21^ and depression is the most common psychiatric disorder in PD, it would be meaningful to distinguish the areas affected purely by depression from those affected by motor symptoms.

Here, we adopted a method of voxel-mirrored homotopic connectivity (VMHC), which measures the FC between each voxel in one hemisphere and its mirrored counterpart in the opposite hemisphere^17^, to investigate the altered interhemispheric connectivity in PD patients. We hypothesized that the depressed and non-depressed PD (DPD and NDPD) patients would exhibit reduced VMHC in different brain regions compared with the healthy controls (HC). Given that depression is associated with abnormal cognitive control from the frontal areas over the subcortical structures, we expected that the prefrontal or limbic regions would be specifically affected in DPD patients besides those motor-related areas affected in both DPD and NDPD.

## Results

### Sample Characteristics

Demographic and clinical characteristics of the participants are presented in Table 1. The three groups did not differ significantly in age, gender, education level and MMSE scores. However, the HDRS scores (*p *< 0.001) were significantly different among the three groups. Moreover, there were no significant differences in PD duration, UPDRS-III, H & Y staging, MoCA, and LED between the DPD and NDPD patients.

### VMHC: Group Differences

**Figure 1:**
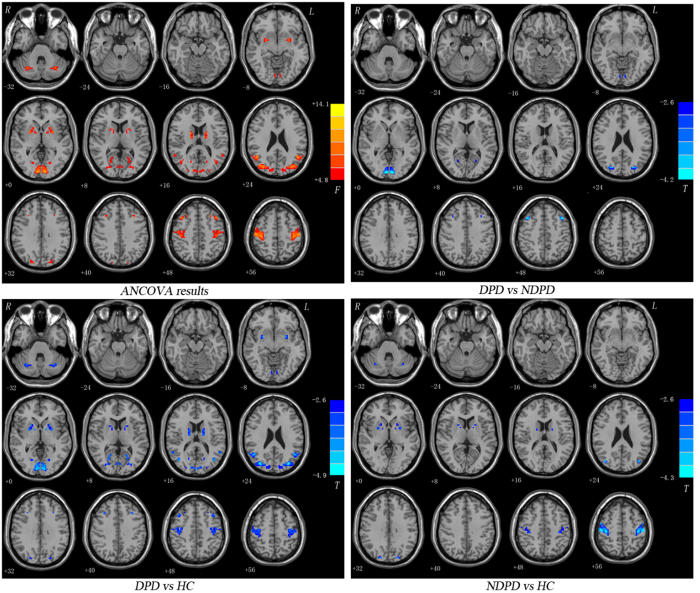
Statistical maps showing VMHC differences in different brain regions between three groups: DPD, NDPD and HC. The threshold for display was set to *p* < 0.01.

Significant differences of the VMHC values among DPD, NDPD and HC groups were observed throughout the frontal, temporal, occipital lobes, subcortical or limbic regions and cerebellum by ANCOVA analysis (Fig. 1; Table 2). The DPD patients showed significantly reduced VMHC values in the dorsolateral prefrontal cortex (DLPFC)^22^,^23^ and calcarine cortex compared with both NDPD and HC (corrected *p *< 0.01). The pooled PD patients (DPD and NDPD) exhibited lower VMHC in the bilateral putamen, middle occipital gyrus (MOG), postcentral gyrus (PoCG), paracentral lobule (PCL) and cerebellum posterior lobe when compared with the HC group. In addition, decreased VMHC of the middle temporal gyrus (MTG) was also observed in the DPD patients relative to the HC. No brain areas showed increased VMHC in either the DPD or the NDPD patients compared with that in the HC. No brain areas showed increased VMHC in the DPD group compared with that in the NDPD.

To test whether these VMHC changes were associated with structural changes, such as gray matter atrophy, we further performed voxel-based morphometry analysis of the gray matter volumes within the brain regions showing significant differences among the three groups. Results showed that the brain areas with decreased VMHC, such as DLPFC, calcarine, and MTG, also showed slightly decreased gray matter volumes in the PD groups compared with that in the HC. However these structural changes were not significant after corrections for multiple comparisons. No correlation was found between the altered VMHC and the altered GMV. More detailed results were shown in the Supplementary Information.

### ROC Analyses between the Patient Groups

**Figure 2:**
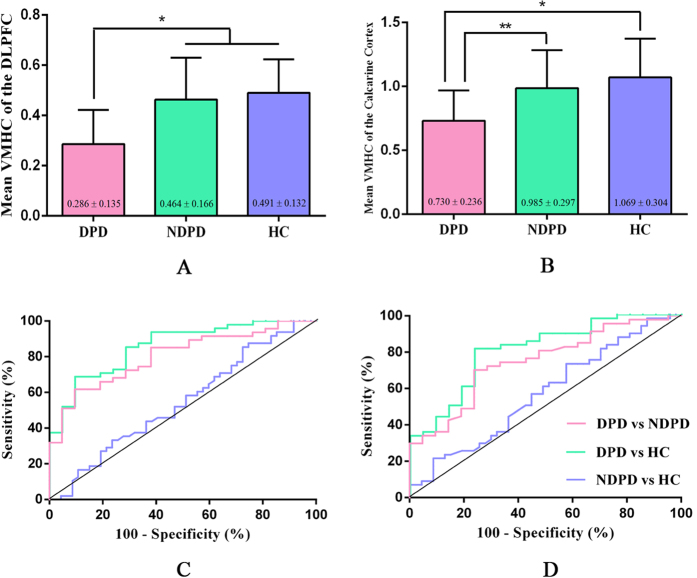
Bar plots representing the mean (and standard deviation) VMHC values of the DLPFC (**A**) and calcarine cortex (**B**) between three groups. Significant differences found between the DPD group and the NDPD or HC group (*p < 0.001, **p = 0.001). ROC analyses by using the mean VMHC values in the DLPFC (C) and calcarine cortex (D) between groups.

Since the DLPFC and calcarine cortex exhibited significant VMHC differences between DPD and NDPD patient groups and between DPD and HC group, they were selected as regions of interest (ROIs). Mean VMHC values were extracted from these two ROIs (Fig. 2A,B) for further receiver operating characteristic curves (ROC) analyses. The results revealed that the areas under the curves (AUC) of the two brain regions were 0.803/0.859 for DLPFC and 0.747/0.806 for calcarine cortex, which indicated that VMHC of the two ROIs could be used to differentiate the DPD from NDPD and from HC. Further analyses showed that the sensitivity and specificity for separating DPD from NDPD and from HC were relatively high (Fig. 2C,D; Table 3).

### Correlations between VMHC and Clinical Variables

Linear correlation analysis was performed between the mean VMHC values within the ROIs (DLPFC and calcarine) and clinical variables of the PD patients. No correlation was found between VMHC of the ROIs and HDRS scores in either DPD or NDPD group, but a significant positive correlation was observed between VMHC of calcarine cortex and MMSE scores in the DPD patients (*r *= 0.474, *p *= 0.030). There was no correlation between VMHC and age, education level, illness duration, UPDRS-III, H&Y, MoCA, and LED.

## Discussion

The present study aimed to investigate the alterations in homotopic FC in PD patients with depression. To our knowledge, this is the first study of using VMHC method to explore the unique association between altered interhemispheric brain synchrony and depression in PD. We found that DPD patients had lower VMHC values in the bilateral DLPFC and calcarine cortex compared with both NDPD and HC groups, and interhemispheric desynchronization in these two brain regions could be used as biomarkers to separate DPD patients from the NDPD and HC. Additionally, when compared to the HC, the DPD and NDPD patients both showed reduced VMHC values mainly in the bilateral putamen, MOG, PoCG, PCL and cerebellum posterior lobe. Furthermore, a significant positive correlation was found between MMSE scores and VMHC values within the calcarine in the DPD patients.

The DLPFC, as a crucial node in the cognitive control network^24^, is involved in cognitive functions, such as attention, working memory and executive control^25^, and is also implicated in emotional control^26^, especially the down-regulation of negative emotion^27^. Based on the Beck’s cognitive model^28^, depressed patients tend to display a cognitive bias towards negative information and away from positive information, which contributes to the sustained depressed mood. These types of high-order cognitive control may have a bi-hemispheric processing advantage based on supporting evidence that interhemispheric coordination is particularly important for the performance of complex tasks and cognitive processes^29^,^30^. The hypoactivity of the DLPFC in depression has been identified by many previous studies, partially consistent with our result. For example, reduced glucose metabolism^31^,^32^, decreased rCBF levels^33^, gray matter reduction^34^ and decreased region homogeneity (ReHo)^35^ in the DLPFC have been reported in patients with depression. Similar results have also been found in DPD patients by Ring HA *et al*., who reported reduced rCBF in the DLPFC in DPD patients^12^. Another resting-state fMRI study of DPD has demonstrated decreased amplitude of low-frequency fluctuations (ALFF) in the DLPFC^36^. Taken together, we speculated that the reduced VMHC within the bilateral DLPFC may underlie the disturbances of the cognitive control systems, which would impede the down-regulation of the negative emotion in DPD patients.

We also observed reduced interhemispheric synchrony between the bilateral calcarine cortex, and the reduced VMHC in the calcarine in the DPD was associated with impaired cognitive functions (decreased MMSE scores). This finding was somewhat unexpected, since the function of calcarine/cuneus has usually been thought to be visual perception and bottom-up attention. We speculated that the decreased VMHC in this “visual area” might indicate impaired sensation and perceptions with depression, or altered cognitive functions, such as the ability to pay attention, which could further mediate the mood regulation processes^37^. During the last decade, converging data from neuroscience and psychology studies with healthy subjects have accrued to discover the multiple high-order cognitive functions of the visual circuits involved in emotion processing, selective attention and their interactions^38^. Meanwhile, studies of MDD also suggested that depression is associated with additional abnormalities besides reduced mood, such as cognitive dysfunction and altered sensory perceptions^37^, and the visual circuits might be involved^39^. Recent studies have shown that visual processing, in as early a stage as the retina, is impaired in depression^6^. The visual evoked potential N1 is sensitive to both depression and antidepressant drugs^37^. Besides these low-level sensory perceptual alterations, the impaired interhemispheric coordination in the visual circuits may also be a potential neural mechanism for the deficits of high-order cognitive functions in depression, such as facial emotion processing. The calcarine cortex, as a primary hub of the visual recognition network^40^, is involved in the perception of facial emotion^40^,^41^.

Antidepressant drugs and depression itself might significantly affect processing of facial expressions of emotion^37^. Structural MRI studies have found that poor visual recall for social stimuli (e.g., facial identification and social scenes on standardized memory scales) in MDD is related to altered cortical thickness and white matter volumes in the lateral surface of the right visual area^39^. Several functional MRI studies have also found decreased activities and VMHC of the visual recognition circuits (i.e., containing the lingual gyrus, middle occipital gyrus, fusiform gyrus and cuneus) in treatment-resistant depression compared with treatment-sensitive depression^39^. Based on these evidences, we speculated that the loss of interest in one’s daily activities and inability to experience pleasure, also known as anhedonia, in DPD might in part be mediated by impaired cognitive functions and sensory abnormalities; where by normal sensory perceptions were no longer paid attention to or present to activate reward circuitry^37^. Although the ROC analyses in the current study demonstrated that VMHC values in the calcarine performed well in differentiating DPD from NDPD and HC groups, and accumulating literatures have recommended the altered visual areas as a possible biomarker in depression^37^,^39^, the visual recognition circuits have not been a robust endophenotype in the depression literature to date and thus this is still a hypothesis that warrants further research.

Additionally, the comparisons between the pooled PD (DPD and NDPD) groups and HC group revealed decreased activities mainly in the striatal-cortical sensorimotor network, including the MOG, PoCG, PCL, putamen and cerebellum. Generally, the cardinal symptoms of PD are thought to be attributed to the dysfunction within this motor circuit^42^.Putamen is a crucial component of the basal ganglia, which plays an important role in this cortico-subcortical circuit, including motor, associative and limbic functions^42^,^43^. At the level of the striatum, the motor circuit is largely centered on the putamen, which receives projections from the motor and somatosensory cortices^42^,^43^, including the primary sensorimotor cortices (PoCG and PCL)^44^. The imbalance in the pathological progression in the striatum, might have led to this desynchronization of putamen in the two hemispheres. At the cortical level, decreased activities in the MOG, PoCG and PCL may result in motor difficulties^16^,^45^ and executive impairment^46^,^47^ in PD patients. Previous studies have also reported decreased putamen-cortical FC^48^ and cortical thinning of the PoCG^49^ and PCL^50^ in PD. Besides, the cerebellum is generally considered to be responsible for motor coordination, balance and speech regulation^51^,^52^ via the cerebello-thalamo-cortical circuit^53^. The alpha-synuclein accumulation has been observed in the cerebellum of PD and can lead to dopamine neuron death^54^. Thus, the reduced interhemispheric FC of cerebellum may indicate poor coordination of the two hemispheres in motor control in PD. Combined with these previous findings, our results suggested that the decreased interhemispheric FC within the cerebello-striatal-cortical circuits might be the main characteristics of PD, which were independent from the non-motor symptoms and were associated with movement disorders.

In addition to the relatively small sample size, the present study has several other limitations. First, the use of medication could be an important confounding factor. To control this factor, we controlled the doses of dopaminergic drugs usage in both of the DPD and NDPD groups, and we evaluated all the patients during their ON state. Second, the brain is not exactly structurally symmetrical. Therefore, we attempted to resolve this issue by smoothing the functional data and normalizing them to a symmetric template^17^. Hence morphometric asymmetry could not account for the reduced VMHC^55^. Third, since the current study mainly focused on the differences of interhemispheric synchrony between PD with and without depression, we adopted only MoCA and MMSE in the evaluation of cognitive functions in PD. More detailed testing than MoCA and MMSE is required to adequately describe participants’ cognitive profile, and to confirm the speculation that bilateral de-synchronization of calcarine may reflect impaired global cognition which mediates the interaction between visual-spatial functions and attentional bias to negative mood stimuli. Finally, VMHC has its own methodological limitations, it can neither be used to investigate the intra-hemispheric connectivity, nor can it reveal which hemisphere is impaired. Hence only limited part of the full picture of the neuropathological changes of brain synchrony in NDPD was revealed in the current study; future researches are warranted to explore both inter- and intra-hemispheric FC in PD patients with depression.

In summary, the decreased VMHC values within the bilateral DLPFC and calcarine cortex may reflect impaired inhibitory control over neural circuits that process emotions and altered sensory perceptions which mediate mood regulations respectively. Decreased activities in these two areas could be used as potential neuroimaging markers to separate DPD patients from NDPD and HC groups. The reduced interhemispheric connectivity of the cerebello-striatal-cortical circuits might be the main characteristics of PD, which were independent from the non-motor symptoms and reflected movement disorders. Our findings might be helpful for the future diagnosis and treatment of depression in PD patients.

## Methods

### Participants

This study was approved by the Medical Research Ethical Committee of Nanjing Brain Hospital (Nanjing, China) in accordance with the Declaration of Helsinki, and written informed consent was obtained from all subjects. Seventy individuals (36 males, 36–71 years) with idiopathic PD were recruited. All of the PD patients fulfilled the UK Parkinson Disease Society Brain Bank Criteria for idiopathic PD^56^.The participants were all right-handed Han Chinese. Patients were excluded if they had (1) cerebrovascular disorders (including history of stroke or head injury, previous neurological surgery and other neurologic diseases); (2) cognitive impairment (Mini-Mental State Examination (MMSE) scores <24); (3) moderate to severe head tremor; (4) antidepressant treatment prior to the beginning of the study or previous psychiatric therapy. Patients on dopamine agonists were also excluded, and the dopamine dosing was stable for at least 4 weeks before and during the study. After enrollment and before the MRI scanning, the patients were followed-up for at least 1 year to confirm the diagnosis.

All the demographic characteristics and clinical symptom ratings were collected before MRI scanning and all patients were in the ON state during the MRI scan. Motor symptoms and PD severity were evaluated by using the Unified PD Rating Scale-motor part III (UPDRS-III) and Hoehn & Yahr (H&Y) staging scale. MMSE and Montreal Cognitive Assessment (MoCA) were used to evaluate cognition. DPD patients were diagnosed with the Diagnostic and Statistical manual of Mental Disorders, Fifth Edition (DSM-V) criteria by an experienced psychiatrist. Afterwards, the severity of depression was quantified with the 17-item Hamilton Depression Rating Scale (HDRS-17). All of the DPD patients diagnosed by DSM-V had HDRS-17 scores higher than 14^57^. Functional images were acquired at the same day of clinical assessment. Moreover, 50 right-handed, age-, gender-, and education-matched HC with no history of neurologic or psychiatric diseases were also included in the research. The same exclusion criteria listed above were applied to the control group.

### Image Acquisition

MRI scanning was performed with a 3.0-T Siemens scanner. Head motion was minimized using foam padding, and head-phones were used to reduce scanner noise. All subjects were instructed to close their eyes, not think of anything particular, and not fall asleep. Axial anatomical images were acquired using a T1 fluid attenuated inversion recovery sequence (TR* *= 2530 ms; TE* *= 3.34 ms; FA* *= 7 degrees; matrix* *= 256 × 192; FOV* *= 256 × 256 mm; slice thickness/gap* *= 1.33/0.5 mm; 128 slices covered the whole brain) for image registration and functional localization. Functional images were subsequently collected in the same slice orientation with a gradient-recalled echo-planar imaging pulse sequence (TR* *= 2,000 ms; TE* *= 30 ms; FA* *= 90 degrees; matrix = 64 × 64, FOV* *= 220 × 220 mm; thickness/gap* *= 3.5/0.6 mm; in-plane resolution* *= 3.4 × 3.4 mm; slice numbers* *= 31). Each functional resting-state session lasted 280 seconds and a total of 140 volumes were obtained in this acquisition sequence.

### Data Preprocessing

Functional images preprocessing was performed using Data Processing Assistant for Resting-State fMRI toolkit (DPARSF, http://www.rest.restfmri.net) which works with Statistical Parametric Mapping software (SPM8, http://www.fil.ion.ucl. ac.uk/spm) on the Matlab platform. The first 5 volumes were discarded to ensure a steady state condition. The remaining images were corrected for slice timing and head motion. Four subjects (2 NDPD and 2 HC) with head motions exceeding 2.5 mm of translation, or 2.5 degrees of rotation, during the whole MRI scans were excluded. The following steps included spatial normalization in the Montreal Neurological Institute (MNI) space using the transformation parameters estimated using an unified segmentation algorithm^58^, re-sampling with a 3 × 3 × 3 mm^3^ resolution, and spatially smoothed with a Gaussian kernel of 6 mm at full-width at half-maximum (FWHM). The resulting fMRI data were linearly trend removed and band-pass (0.01−0.08 Hz) filtered to reduce low-frequency drift and high-frequency noise. There was no difference in mean head motion (*P *> 0.05). Several sources of spurious variance were removed, including the six motion parameters obtained by head-motion correction, signals from the whole brain, ventricular system, and white matter^59^. Subsequently, the images of each subject were registered to a study-specific symmetric MNI template and were then used to compute the VMHC.

### Voxel-Mirrored Homotopic Connectivity

The VMHC was calculated with REST software (http://www.resting-fmri.sourceforge.net). Briefly, the homotopic FC for each subject was computed as the Pearson correlation coefficient between each voxel’s residual time series and that of its symmetrical interhemispheric counterpart. Correlation values were then Fisher z-transformed to improve normality. The resultant values constitute the VMHC and were used for group analyses. The details of VMHC obtainment were described in a previous study^17^.

### Statistical Analysis

Differences among the three groups (DPD, NDPD and HC) in terms of demographic and clinical variables were performed by one-way analysis of variance (ANOVA), the Pearson χ^2^ test, or the Student t test, as appropriate. All tests were two-tailed and *p *< 0.05 was considered statistically significant. All analyses were conducted using SPSS 18.0.

Voxel-based comparisons of the entire VMHC maps were processed with REST software. Statistical tests across groups were performed using a voxel-based, one-way analysis of covariance (ANCOVA), with age, gender and education level as covariates, followed by post-hoc two-sample t tests. The ANCOVA result was corrected with a cluster-level significance threshold of *p *< 0.01 (voxel-level *p *< 0.01 and cluster size > 42 voxels determined by a Monte Carlo simulation, see AlphaSim in AFNI http://afni.nimh.nih.gov/pub/dist/doc/manual/AlphaSim.pdf; parameters: single voxel *p *= 0.01, 6-mm FWHM, within the unilateral hemispheric gray-matter mask). The post-hoc two-sample t tests were conducted within a mask showing significant differences obtained from the ANCOVA analysis, with corrections (voxel-level *p *< 0.01; cluster size > 12 voxels; determined by a Monte Carlo simulation resulted in a cluster-level significance threshold of *p *< 0.01).

Brain regions exhibiting significant differences both between the DPD and NDPD groups and between DPD and HC groups were selected as ROIs. Mean VMHC values were extracted within each of these ROIs for further ROC analyses. Furthermore, we computed Pearson correlation coefficients between the extracted VMHC values within these ROIs and the clinical variables of DPD patients, using SPSS 18.0, and the significance level was set at *p *< 0.05 (two-tailed).

## Additional Information

**How to cite this article**: Zhu, Y., *et al*. Impaired interhemispheric synchrony in Parkinson’s disease with depression. *Sci. Rep*. **6**, 27477; doi: 10.1038/srep27477 (2016).

## Supplementary Material

Supplementary Information

## Figures and Tables

**Table 1:** Demographic and clinical characteristics of the participants.

Variables	DPD	NDPD	HC	*p* Value
	(n = 21)	(n = 47)	(n = 48)	
Age, years	58.10 ± 7.53	57.70 ± 7.03	57.67 ± 5.59	0.967^a^
Gender, male/female	9/12	26/21	23/25	0.593^b^
Education, years	11.05 ± 3.07	10.89 ± 3.29	11.56 ± 4.86	0.703^a^
Disease duration, years	5.43 ± 2.77	6.24 ± 3.35	NA	0.335^c^
HDRS	20.19 ± 4.62	6.87 ± 3.10	2.15 ± 2.37	<0.001^1,2,3^
UPDRS-III	28.29 ± 13.16	26.46 ± 13.35	NA	0.602^c^
H & Y	1.45 ± 0.59	1.75 ± 0.65	NA	0.064^b^
MMSE	28.67 ± 1.11	28.60 ± 1.72	28.98 ± 2.35	0.604^a^
MoCA	25.19 ± 2.79	25.09 ± 3.44	NA	0.902^c^
LED, mg/day	502.68 ± 354.98	478.83 ± 362.77	NA	0.801^c^

Values are represented as the mean ± standard deviation. For comparisons of demographics:

^a^p values were obtained by one-way analysis of variance (ANOVA) tests.

^b^*p* value for the gender difference and H&Y staging was obtained by chi-square test.

^c^*p* values were obtained by two-sample *t* test. Comparison of HDRS scores among the three groups was performed using a separate one-way ANOVA test, and post-hoc tests were then performed using the *t* test.

^1,2,3^Post-hoc paired comparisons showed significant group differences between DPD versus NDPD, DPD versus HC, and NDPD versus HC, respectively. *p *< 0.05 was considered significant. HDRS = Hamilton Depression Rating Scale; UPDRS-III = Unified Parkinson’s Disease Rating Scale-motor part III; H & Y = Hoehn & Yahr staging scale; MMSE = Mini-Mental State Examination; MoCA = Montreal Cognitive Assessment; LED = levodopa equivalent dose; NA, not applicable.

**Table 2:** Regions showing significant differences in VMHC between groups.

Brain Regions(AAL)	Cluster Size	MNI Coordinates			T Value
		X	Y	Z	
DPD* *< NDPD					
Middle frontal gyrus	28	±39	18	48	−4.1285
Calcarine cortex	66	±9	−93	0	−4.1567
Superior occipital gyrus	24	±24	−81	27	−3.7108
DPD* *< HC					
Middle frontal gyrus	39	±39	18	45	−4.1467
Calcarine cortex	94	±6	−90	0	−4.4562
Putamen	107	±27	3	−6	−4.0595
Middle occipital gyrus	118	±33	−81	24	−4.8243
Middle temporal gyrus	61	±57	−54	18	−4.4601
Postcentral gyrus	148	±48	−12	51	−4.0856
Paracentral lobule	50	±6	−33	69	−3.7651
Cerebellum posterior lobe	43	±21	−66	−30	−4.7630
NDPD* *< HC					
Putamen	59	±21	3	−6	−4.0371
Middle occipital gyrus	13	±42	−75	21	−4.1968
Superior occipital gyrus	12	±18	−87	36	−3.5644
Postcentral gyrus	164	±51	−21	57	−4.2159
Paracentral lobule	72	±12	−18	75	−3.9608
Cerebellum posterior lobe	18	±21	−69	−27	−3.8826

A corrected threshold of *p *< 0.01 corrected by Monte Carlo; cluster size is in mm3; two-sample *t* tests with age, gender, and education level as covariates were performed to test the VMHC differences between groups. MNI = Montreal Neurological Institute.

**Table 3:** ROC analyses for differentiating different groups.

Brain regions	AUC	*p* value	95% CI	Sensitivity	Specificity	Cut-off point
DLPFC						
* Seprating DPD from NDPD*	0.803	<0.001	0.698 − 0.909	0.905 (19/21)	0.617 (29/47)	0.441^a^
* Seprating DPD from HC*	0.859	<0.001	0.768 − 0.951	0.905 (19/21)	0.688 (33/48)	0.439
* Seprating NDPD from HC*	0.546	0.443	0.429 − 0.662	0.277 (13/47)	0.854 (41/48)	0.376
Calcarine cortex						
* Seprating DPD from NDPD*	0.747	0.001	0.624 − 0.869	0.762 (16/21)	0.702 (33/47)	0.848
* Seprating DPD from HC*	0.806	<0.001	0.694 − 0.917	0.762 (16/21)	0.813 (39/48)	0.827
* Seprating NDPD from HC*	0.566	0.267	0.450 − 0.682	0.426 (20/47)	0.729 (35/48)	0.924

^a^By this cut-off point, the VMHC values of the DLPFC could correctly classify 19 of 21 DPD patients and 29 of 47 NDPD patients, resulted in a sensitivity of 90.5% and a specificity of 61.7%. The means of other cut-off points were similar. AUC = area under the curve; CI = confidence interval.
