# Supplementary material for: Impaired interhemispheric synchrony in Parkinson’s disease with depression
Source: Sci Rep. 2016 Jun 6;6:27477. doi: 10.1038/srep27477 (PMC4893739; doi:10.1038/srep27477)
Supplement: Supplementary Information [file srep27477-s1.doc]

**Impaired interhemispheric synchrony in**

**Parkinson's disease with depression**

Yajing Zhu^1,+^, Xiaopeng Song^2,+^, Mingze Xu^2^, Xiao Hu^1^, Erfeng Li^1^, Jiajia Liu^1^, , Yonggui Yuan^4^, Jiahong Gao^2,3^, Weiguo Liu^1^,**^*^**

^1^Department of Neurology, Affiliated Brain Hospital of Nanjing Medical University, Nanjing 210029, China

^2^Department of Biomedical Engineering, College of Engineering, Peking University, Beijing 100871, China

^3^Center for MRI Research, Beijing City Key Lab for Medical Physics and Engineering, McGovern Institution for Brain Research, Peking University, Beijing 100871, China

^4^Department of Psychiatry and Psychosomatics, Affiliated ZhongDa Hospital of Southeast University, The Institute of Neuropsychiatry of Southeast University, Nanjing 210009, China

**^*^**Corresponding author: Dr. Weiguo Liu; Department of Neurology, Affiliated Brain Hospital of Nanjing Medical University, Nanjing 210029, China; Phone: +86-13914725569; Email: wgliubh@sina.com

^+^These authors contributed equally to this work

Supplementary Information:

Regional grey matter volume differences (GMV) were calculated on modulated data using *cat12* (also known as *VBM*, a toolbox of *SPM*, [*http://www.neuro.uni-jena.de/vbm/*](http://www.neuro.uni-jena.de/vbm/)). In order to correct for the whole brain size, we included the total intracranial volume (TIV) as a covariate, as well as gender, age and years of education. Two-sample t tests were performed within the mask of brain regions that showed significant differences of VMHC among the three groups. The results of two-sample t tests between DPD and HC groups (Supplementary Figure 1), between NDPD and HC groups (Supplementary Figure 2), as well as between DPD and NDPD groups (Supplementary Figure 3) were shown below. None of these clusters survived the corrections for multiple comparisons.

|  |  | | | MNI | Coordinates |  |  |
| --- | --- | --- | --- | --- | --- | --- | --- |
| Brain Regions(AAL) | | Cluster Size | X | | Y | Z | T Value |
| **DPD < HC** | |  |  | |  |  |  |
| Temporal_Inf_R | | 55 | 52.5 | | − 39 | -27 | 2.9876 |
| Hippocampus_R | | 169 | 27 | | − 25.5 | -12 | 3.0151 |
| ParaHippocampal_L  Putamen_R  Thalamus_R  Occipital_Mid_L  Postcentral_R  Precuneus_R | | 74  30  66  123  186  60 | -28.5  34.5  7.5  -37.5  36  9 | | − 43.5  -4.5  -24  -78  -31.5  -61.5 | -4.5  3  16.5  36  49.5  45 | 3.3508  2.7322  4.266  2.9582  3.1402  2.8821 |
| **NDPD < HC** | |  |  | |  |  |  |
| Temporal_Inf_L | | 78 | -57 | | -10.5 | -36 | 3.6046 |
| Frontal_Inf_Orb_R | | 210 | 33 | | 16.5 | -21 | 4.4268 |
| Fusiform_L | | 150 | -39 | | -63 | − 16.5 | 4.1816 |
| Fusiform_R | | 802 | 28.5 | | -57 | -12 | 4.8379 |
| Rectus_R | | 197 | 9 | | 43.5 | -16.5 | 3.7332 |
| Temporal_Sup&Mid_R  Frontal_Mid_Orb_R  Frontal_Mid_Orb_L | | 393  90  236 | 61.5  37.5  -22.5 | | − 12  43.5  46.5 | -9  -10.5  -10.5 | 4.6773  3.6509  3.8583 |
| Temporal_Mid_L | | 205 | -54 | | -49.5 | -15 | 3.8336 |
| Lingual_R  Fusiform_L  Lingual_L  Temporal_Sup_L  Temporal_Sup_R  Frontal_Sup_Medial_L  Cingulum_Mid_R  Supp_Motor_Area_R | | 328  70  145  940  634  287  63  183 | 4.5  -27  -31.5  -55.5  54  -1.5  9  7.5 | | -73.5  -72  -54  -13.5  -13.5  57  36  13.5 | −6  -9  -10.5  7.5  10.5  22.5  28.5  45 | 5.3833  4.135  3.9779  5.1333  4.5903  4.1512  3.794  3.9888 |
| **NDPD > HC** | |  |  | |  |  |  |
| Putamen_R | | 77 | 25.5 | | -6 | -1.5 | 3.5959 |
| Putamen_L | | 147 | -25.5 | | -9 | 0 | 4.0859 |
| Postcentral_R | | 125 | 16.5 | | -49.5 | 70.5 | 4.9816 |
| **NDPD > DPD**  Fusiform_R  Lingual_L  Putamen_R  Occipital_Mid_L  Putamen_L  **DPD > NDPD**  ParaHippocampal_R  Lingual_L  Temporal_Mid_L  Parietal_Inf_L | | 29  63  94  74  132  353  147  102  33 | 37.5  -15  24  -28.5  -22.5  30  -25.5  -61.5  -51 | | -12  -43.5  -3  -88.5  -4.5  -37.5  -48  -52.5  -43.5 | -39  -9  9  7.5  7.5  -12  -4.5  4.5  51 | 3.7203  3.6691  3.4932  3.8816  3.7796  4.1175  3.9212  3.9289  3.7887 |

**Supplementary Table 1. Brain regions showing differences in GMV between groups.** two-sample t tests with age, gender, years of education, TIV as covariates were performed to test the GMV differences between groups (*p* < 0.001 with cluster size > 20 voxels, uncorrected); The unit of cluster size is in #voxel.


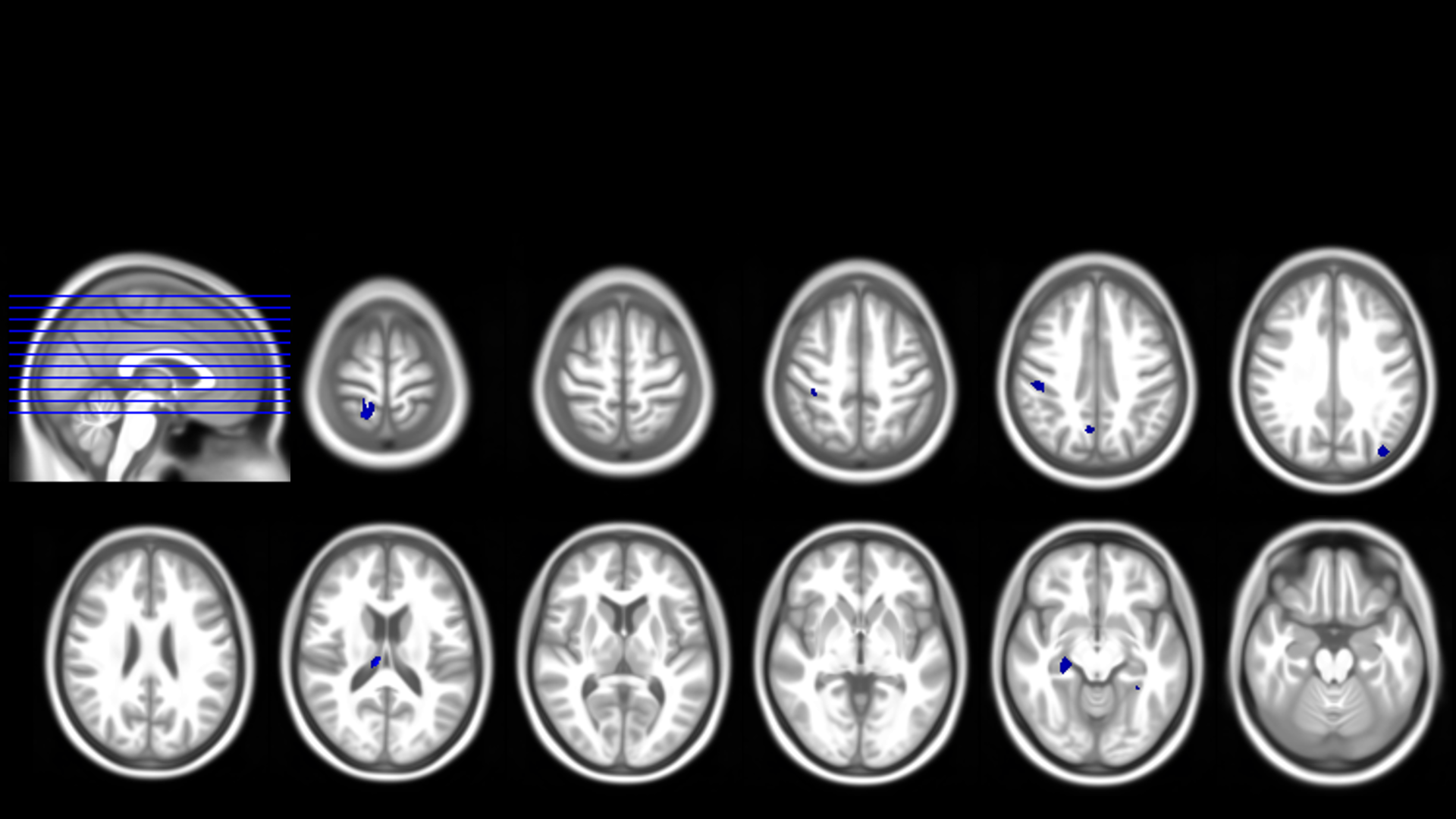


**Supplementary Figure 1.** GMV differences between DPD and HC (*p* < 0.001 with cluster size > 20 voxels, uncorrected). Blue indicates GMV in the DPD is smaller than that in the HC. No greater GMV was found in the DPD than that in the HC.


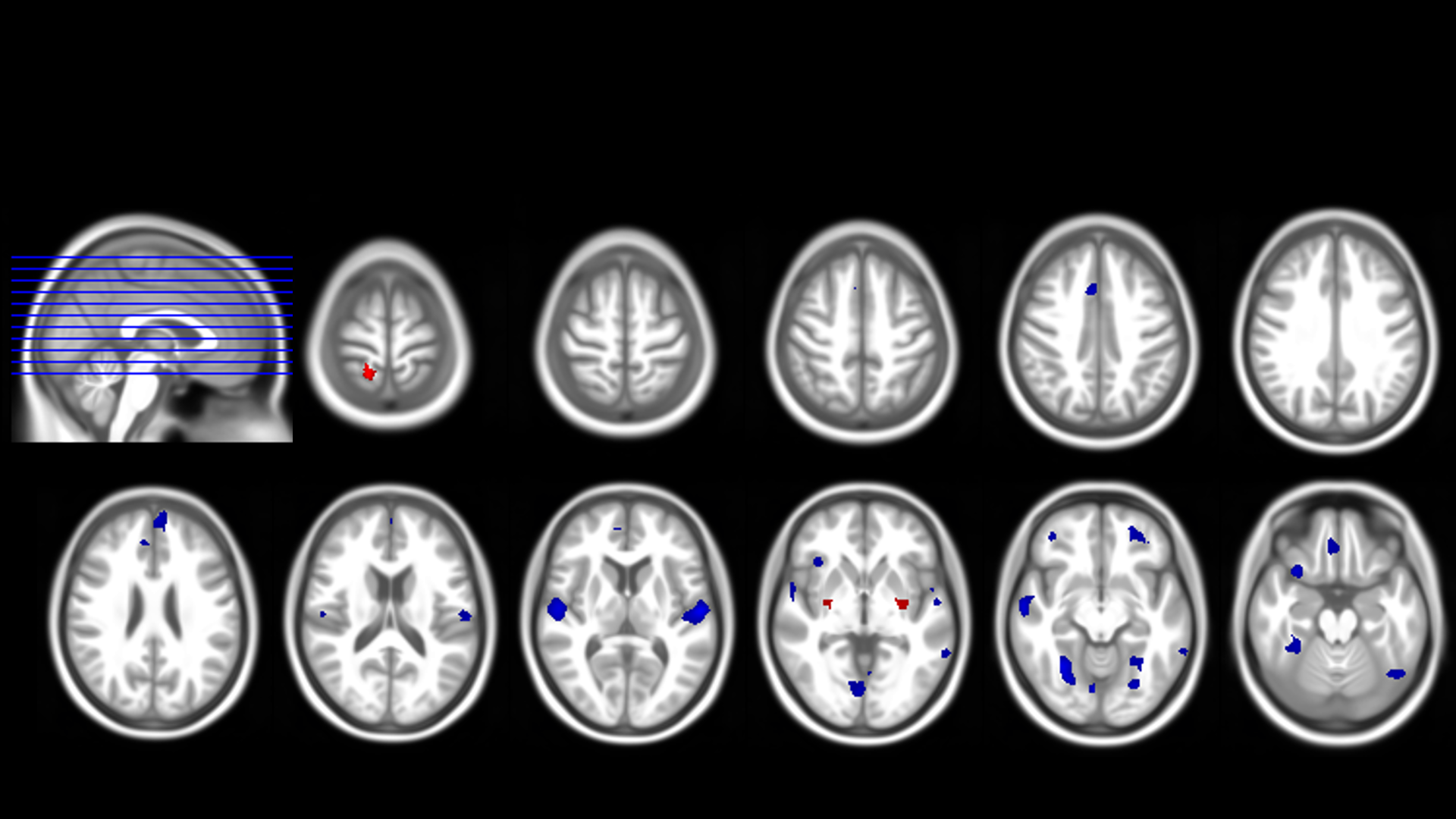


**Supplementary Figure 2.** GMV differences between NDPD and HC (*p* < 0.001 with cluster size > 20 voxels, uncorrected). Blue indicates GMV in the NDPD is smaller than that in the HC. Red indicates GMV in the NDPD is greater than that in the HC.


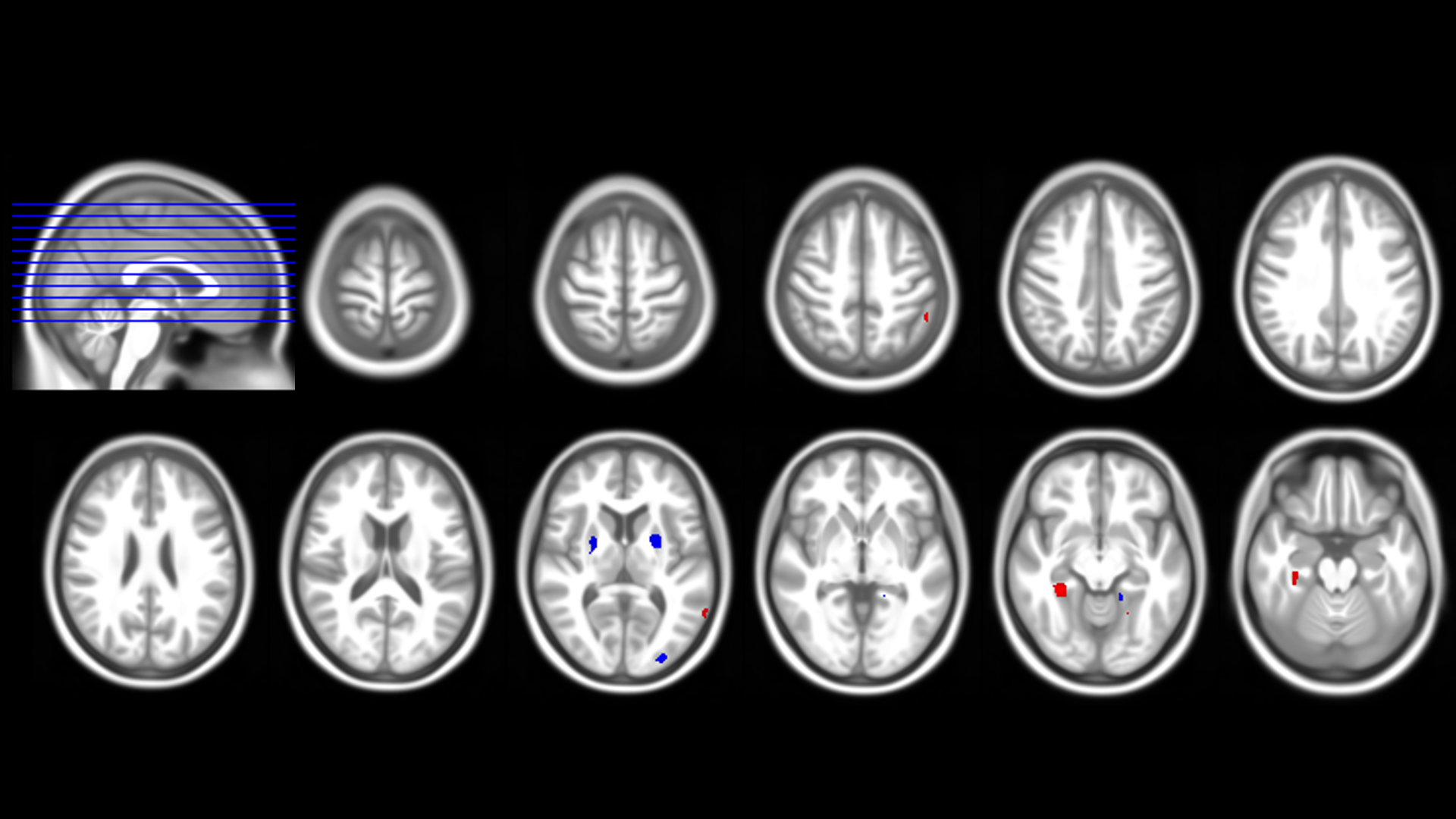


**Supplementary Figure 3.** GMV differences between DPD and NDPD (*p* < 0.001 with cluster size > 20 voxels, uncorrected). Blue indicates GMV in the DPD is smaller than that in the NDPD. Red indicates GMV in the DPD is greater than that in the NDPD.
